# Exposure to diesel exhaust particles increases susceptibility to invasive pneumococcal disease

**DOI:** 10.1016/j.jaci.2019.11.039

**Published:** 2020-04

**Authors:** Rebecca K. Shears, Laura C. Jacques, Georgia Naylor, Lisa Miyashita, Shadia Khandaker, Filipa Lebre, Ed C. Lavelle, Jonathan Grigg, Neil French, Daniel R. Neill, Aras Kadioglu

**Affiliations:** a Bacterial Pathogenesis and Immunity Group, Institute of Infection and Global Health, University of Liverpool, Liverpool, United Kingdom; b Centre for Genomics and Child Health, Blizard Institute, Queen Mary University of London, London, United Kingdom; c Adjuvant Research Group, School of Biochemistry and Immunology, Trinity Biomedical Sciences Institute, Trinity College Dublin, Dublin, Ireland; d Microbial Evolution, Genomics and Adaptation Group, Institute of Infection and Global Health, University of Liverpool, Liverpool, United Kingdom

**Keywords:** *Streptococcus pneumoniae*, pneumococcus, pneumonia, pneumococcal disease, particulates, pollution, BAL, Bronchoalveolar lavage, BMDM, Bone marrow–derived macrophage, CFU, Colony-forming unit, DEP, Diesel exhaust particle, FITC, Fluorescein isothiocyanate, FSC, Forward scatter, GFP, Green fluorescent protein, IPD, Invasive pneumococcal disease, OPKA, Opsonophagocytic assay, UK, United Kingdom

## Abstract

**Background:**

The World Health Organization estimates that air pollution is responsible for 7 million deaths per annum, with 7% of these attributable to pneumonia. Many of these fatalities have been linked to exposure to high levels of airborne particulates, such as diesel exhaust particles (DEPs).

**Objectives:**

We sought to determine whether exposure to DEPs could promote the progression of asymptomatic nasopharyngeal carriage of *Streptococcus pneumoniae* to invasive pneumococcal disease.

**Methods:**

We used mouse models and *in vitro* assays to provide a mechanistic understanding of the link between DEP exposure and pneumococcal disease risk, and we confirmed our findings by using induced sputum macrophages isolated from healthy human volunteers.

**Results:**

We demonstrate that inhaled exposure to DEPs disrupts asymptomatic nasopharyngeal carriage of *S pneumonia*e in mice, leading to dissemination to lungs and blood. Pneumococci are transported from the nasopharynx to the lungs following exposure to DEPs, leading to increased proinflammatory cytokine production, reduced phagocytic function of alveolar macrophages, and consequently, increased pneumococcal loads within the lungs and translocation into blood. These findings were confirmed by using DEP-exposed induced sputum macrophages isolated from healthy volunteers, demonstrating that impaired innate immune mechanisms following DEP exposure are also at play in humans.

**Conclusion:**

Lung inhaled DEPs increase susceptibility to pneumococcal disease by leading to loss of immunological control of pneumococcal colonisation, increased inflammation, tissue damage, and systemic bacterial dissemination.

## Graphical abstract

**Figure:**
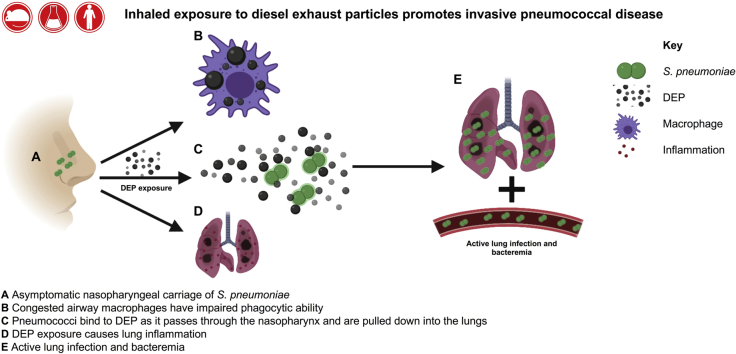

Air pollution is a major environmental risk to health, with outdoor air pollution estimated to cause 4.2 million premature deaths worldwide in 2016 and household air pollution adding a further 3.8 million deaths to the pollution-associated death toll.^1^ Of these deaths, 91% occurred in low- and middle-income countries, with respiratory (eg, asthma, chronic obstructive pulmonary disease, lung cancer, and lower respiratory tract infections) and cardiovascular diseases contributing to the largest disease burden.^2^, ^3^, ^4^, ^5^, ^6^, ^7^, ^8^, ^9^, ^10^, ^11^, ^12^, ^13^, ^14^, ^15^, ^16^, ^17^, ^18^, ^19^ Ambient air pollution can originate from natural sources, such as dust storms or forest fires; however, the vast majority of air pollution is from anthropogenic sources such as heat and power generation, industrial activities, and motor vehicles (both gasoline and diesel).^20^ Carbon monoxide, nitrogen dioxide, and sulphur dioxide are responsible for many of the damaging effects of air pollution; however, particulate matter (PM) is also associated with a wide range of health defects.^21^ PM is often divided into 2 categories: PM10 (PM with an aerodynamic diameter between 2.5 and 10 μm) and fine PM (PM with an aerodynamic diameter of ≤2.5 μm).^21^ Both types of PM are associated with increased hospital admissions for pneumonia in population studies, particularly in industrialized countries.^2^, ^3^, ^4^, ^5^

*Streptococcus pneumoniae* is the most common cause of community-acquired pneumonia, with mortality rates of more than 20% in patients with accompanying septicemia.^22^ The pneumococcus is also the leading cause of infectious disease deaths in children under the age of 5 years worldwide.^22^, ^23^, ^24^ *S pneumoniae* is part of the normal flora of the human nasopharynx; however, if the bacterium is able to gain access to normally sterile sites such as the lungs, blood, and meninges, it has potential to cause life-threatening disease such as pneumonia, septicemia, and meningitis.^22^ Little is known about the conditions that allow the bacterium to progress from a harmless commensal to a potentially life-threatening pathogen; however, exposure to high levels of pollution is a known risk factor for development of invasive pneumococcal disease (IPD).^2^^,^^3^^,^^25^, ^26^, ^27^, ^28^

We have previously identified a link between high levels of airborne dust and epidemics of pneumococcal meningitis in the Sahel region of Sub-Saharan Africa.^29^ In the developing world, agricultural and household pollution are the major sources of airborne PM; however, traffic and industrial pollution are increasingly recognized as the biggest contributors to air pollution in developed and industrialized countries.^1^^,^^5^^,^^21^^,^^30^^,^^31^ In particular, there is a strong body of evidence to suggest that exposure to diesel exhaust particles (DEPs), which are often used as a surrogate for traffic-related pollution, is linked to cardiovascular defects in addition to respiratory disease.^32^, ^33^, ^34^, ^35^ DEP inhalation has been reported to increase lung inflammation and reduce lung function in rodent studies.^36^^,^^37^ DEP exposure has also been shown to aggravate allergic airway inflammation and exacerbate asthma-like inflammation.^38^, ^39^, ^40^, ^41^, ^42^ Additionally, DEP exposure has been linked to impaired clearance of respiratory pathogens. Short-term exposure to ambient concentrations of diesel engine emissions impaired clearance of *Pseudomonas aeruginosa* and led to increased lung inflammation and pathology in mouse models,^43^ whereas intratracheal instillation of DEPs has been shown to impair lung clearance of *Listeria monocytogenes* in rats.^44^ DEP exposure has also been shown to enhance influenza infection of respiratory epithelial cells *in vitro*, leading to increased oxidative stress.^45^

Here, we provide mechanistic data to explain the association between DEP inhalation and pneumococcal disease by using murine models and *in vitro* assays. We show that upon inhalation of DEPs, alveolar macrophages become congested with PM, which significantly reduces their phagocytic ability, thereby impairing bacterial clearance and increasing bacterial loads within the lungs. DEP inhalation stimulates a proinflammatory environment, which is exacerbated by the high bacterial load. This highly inflammatory environment caused by the combination of particulate and high bacterial load is likely to cause tissue damage, allowing the bacterium access to the bloodstream. We also provide data to suggest that DEPs may provide metabolites to sustain the survival of pneumococci within the lungs.

## Methods

### Bacteria and particulate

The mouse-virulent serotype 2 *S pneumoniae* strain D39 (NCTC 7466) was cultured on blood agar base supplemented with 5% (vol/vol) defibrinated horse blood or in brain heart infusion broth containing 20% (vol/vol) FBS. Bacteria were identified as pneumococci by α-hemolysis on blood agar and by optochin sensitivity. Bacterial suspensions were standardized for inoculation and stored at –80°C. When required, suspensions were thawed at room temperature and bacteria were harvested by centrifugation and suspended in PBS. DEPs (SRM 2975) were purchased from the National Institute of Standards and Technologies (https://www-s.nist.gov/srmors/view_cert.cfm?srm=2975).

### Mice

Female CD1, C57BL/6, or BALB/c mice (Charles River, Margate, United Kingdom [UK]) were maintained in individually ventilated cages at 22°C ± 1°C and 65% humidity with a 12-hour light-dark cycle. Mice were acclimatized for 1 week before use and had free access to food and water. All procedures were carried out on age-matched mice aged 6 to 8 weeks or older. All experimental protocols were approved and performed in accordance with the regulations of the Home Office Scientific Procedures Act (1986), Project license P86De83DA, and the University of Liverpool Animal Welfare and Ethical Review Body.

### Mouse model of pneumococcal carriage

Mice were lightly anesthetized with a mixture of O_2_ and isoflurane and exposed once daily to either 80 μg of DEPs dissolved in 40 μL of PBS or PBS alone via the intranasal route for the duration of the experiment, unless otherwise stated. After 3 exposures, mice were anesthetized before intranasal infection with 1 × 10^5^ colony-forming units (CFU) of D39 in 10 μL of PBS. Control mice were treated with 10 μL of PBS only. Mice were humanely killed 1, 4, 7, or 10 days postinfection. The nasopharynx and lungs were removed from each mouse and blood was collected in heparin tubes. Bacterial viable counts were determined as described previously.^46^ Lung cell suspensions were stored at –80°C following erythrocyte lysis and supernatants were also stored at –80°C until required. For histologic analysis, lungs of PBS- or DEP-exposed mice were fixed overnight in neutral buffered formalin (10% formalin) followed by 95% ethanol and processed using standard histologic techniques. Six-micron frontal sections of the lungs were stained with hematoxylin and eosin and imaged using an Aperio CS2 Slide Scanner (Leica, Milton Keynes, UK). Apeiro Image Scope v12.3.2.8013 was used to analyze images.

### Mouse model of pneumococcal pneumonia

Female BALB/c mice were exposed daily to 80 μg of DEPs in 40 μL of PBS as described earlier in this article. After 3 intranasal exposures, mice were intranasally infected with 1 × 10^6^ CFU of D39 in 50 μL of PBS to induce pneumococcal pneumonia. Mice were culled at either 24 or 72 hours postinfection and lung and blood viable counts were calculated as described earlier.

### Flow cytometry analysis

Single-cell suspensions from the lungs were stained as follows: panel 1, CD45–fluorescein isothiocyanate (FITC) (30-F11, eBiosciences, Thermo Scientific, Rugby, UK), CD4-APC-CY7 (RM4-5, BD Biosciences, Wokingham, UK), and FOXP3-Pacific blue (MF-14, Biolegend, London, UK); and panel 2, CD45-FITC, GR1-PE-CY7 (RB6-8C5, BD Biosciences), CD11b-PE (M1/70, Biolegend), and F4/80-Pacific blue (T45-2342, BD Biosciences). Acquisition was carried out by using a FACSCanto flow cytometer (BD Biosciences), and the analysis was performed using Flowjo X (Tree Star, Ashland, Ore). Fluorescent minus 1 control for each of the included antibodies were used to validate results.

### Cytokine analysis

Quantification of TNF-α, IFN-γ, IL-1β, keratinocyte chemoattractant/human growth-regulated oncogene (KC/GRO), macrophage inflammatory protein 2 (MIP-2), and IL-6 in lung homogenates was performed by using a U-Plex cytokine assay (Meso Scale Discovery, Rockville, Md) according to the manufacturer’s instructions. Day 1 lung homogenates were diluted 2-fold, whereas day 4 and 7 lung homogenates were diluted 10-fold before the assay was run.

### Giemsa staining of murine alveolar macrophages

Mice were humanely killed at various time points and airway macrophages were isolated by bronchoalveolar lavage (BAL) in 2 mL of PBS, as described previously.^47^ The BAL fluid was fixed onto microscope slides by using a Cytospin 4 (Thermo Scientific) at 1000 rpm. Slides were fixed with Kwik-Diff Reagent 1 for 5 seconds, followed by eosin and methylene blue (all Thermo Scientific) for 5 seconds and then washed in distilled water. Slides were imaged by using an Olympus BH-2 microscope.

### Quantification of DEP congestion

Giemsa-stained airway macrophages were scored according to how heavily congested with DEPs they were. The high contrast between DEP- and Giemsa-stained alveolar macrophages allowed simple visual discrimination of DEPs and cellular contents. Cells were scored as characterized by low congestion (<30% cytoplasmic coverage), medium congestion (30% to 60% cytoplasmic coverage), or high congestion (>60% cytoplasmic coverage). The scoring system was applied to 25 macrophages per mouse over several slides. The data displayed show the mean percentage of macrophages with no visible DEP congestion and low, medium, and high DEP congestion for 3 to 5 mice.

### Cell culture

J774.2 murine macrophages were maintained in Dulbecco’s modified Eagle’s medium supplemented with 5% FBS as previously described.^47^ Cell lines were confirmed to be *Mycoplasma* negative before use.

### J774.2 OPKAs

Opsonophagocytic killing assays (OPKAs) were performed with J774.2 cells as described previously,^47^ with the following modifications: cells were incubated with 20 μg of DEPs per well (40 μL of 0.5 mg/mL DEP solution) for 2 hours before addition of opsonized pneumococci. DEP solution was sonicated before use in the OPKAs.

### Human sputum macrophage phagocytic killing assays

Participants were given inhaled salbutamol (albuterol) via a multidose inhaler and large volume spacer (Volumatic, Allen and Hanbury, Uxbridge, UK). Induced sputum cells were then obtained after inhalation of nebulized 4.5% hypertonic saline via a Multisonic Profi nebulizer (Schill, Laichingen, Germany) for a maximum of 20 minutes, as described by Kulkarni et al.^48^ Whole induced sputum samples were diluted 1:1 with PBS, vortexed, and centrifuged for 10 minutes at 3000 rpm. The supernatant was discarded, and mucolysis carried out with the addition of 0.1% dithiothreitol (Sigma-Aldrich, Gillingham, UK) for 10 minutes, shaking at room temperature. Samples were filtered and centrifuged for 10 minutes at 10,000 rpm. The cell pellet was subsequently resuspended in 1 mL of PBS (2% FCS) and enriched for airway macrophages by incubation with 60 μL of monocyte enrichment cocktail (RosetteSep, Stem Cell Technologies, Cambridge, UK) and 40 μL of erythrocytes. The airway macrophages were isolated by Ficoll separation and suspended into chamber well slides overnight. The airway macrophages were subsequently exposed to PBS or DEPs (10 μg/mL) for 2.5 hours before washing and addition of *S pneumoniae* D39 (OD 0.5) for 2 hours. The chambers were removed and slides washed before staining (Hemacolour, Merck Millipore, UK). The cells were imaged under light microscopy (20 macrophages per condition for each volunteer) and analyzed for mean bacterial load per macrophage (Image J software).

### Bone marrow derived macrophages assays

Murine bone marrow–derived macrophages (BMDMs) were isolated from female C57BL/6 mice and cultured as described previously.^49^ On day 6, cells were plated at 0.8 × 10^6^ per well for use in assays. On day 7, the cells were treated with medium or DEPs (4, 20, or 100 μg). After 24 hours, the cells were washed with PBS to remove the DEPs and the medium was replaced. On day 13, the cells were stimulated with D39 (8 × 10^6^ per well). After 24 hours, supernatants were collected for cytokine quantification. The cells were lysed and total protein was measured by using a Pierce bicinchoninic acid protein assay kit (Thermo Scientific) according to the manufacturer’s instructions. TNF-α and IL-6 production were measured by ELISA (Biolegend) and normalized to total protein content.

### *In vitro* growth of pneumococci

For the *in vitro* growth experiments, pneumococci were grown in M9 minimal medium supplemented with 0.1% or 1% DEPs. Viable counts were performed over time (2 to 7 hours) and were compared to the viable counts for pneumococci grown in medium without supplement.

### Investigating the adherent properties of DEPs

Green fluorescent protein (GFP)-tagged D39 (D39-GFP) was incubated with DEPs for 20 minutes on an over-end rotator. Following centrifugation at 400 × g for 5 minutes, unbound pneumococci were removed (supernatant) and the pellet (containing DEPs and bound pneumococci) was washed twice with PBS. The DEP pellet was resuspended in PBS, and FITC fluorescence was measured by using flow cytometry. Adherence was defined as a shift in forward scatter (FSC)-high GFP events.

### Scanning electron microscopy

Samples were prepared for scanning electron microscopy as follows. The bacteria were incubated with DEPs for 15 minutes before washing in PBS (as described earlier) and fixation in 4% (wt/vol) paraformaldehyde and 2.5% (wt/vol) glutaraldehyde in 0.1M phosphate buffer (pH 7.4). After another wash step, the bacteria and DEPs were pipetted onto poly-l-lysine–coated glass coverslips before staining with reduced osmium (2% osmium tetroxide in distilled water + 1.5% potassium ferrocyanide in 0.1M PB) followed by 2% (wt/vol) aqueous uranyl acetate. Dehydration was performed in graded ethanol followed by hexamethyldisilazane. To prevent precipitation artifacts the samples were washed copiously with double-distilled water between each staining step.

Coverslips were attached to scanning electron microscopy stubs and then sputter-coated with 10 nm of AuPd (QuorumTechnologies Q150T. Lewes, UK). Secondary electron scanning electron microscopy imaging was performed in a Quanta 250 FEG (FEI, Hillsboro, Ore) at low vacuum (0.53 Torr) with an acceleration voltage of 5 kV.

### Statistical analysis

All statistical analysis was carried out using GraphPad Prism 7 software (GraphPad Inc, La Jolla, Calif). The Mann-Whitney *U* test and ANOVA with Kruskall-Wallis posttest were performed when comparing 2 and 3 experimental groups, respectively. Error bars represent the SEM.

## Results

### Inhaled DEP exposure promotes IPD

**Fig 1:**
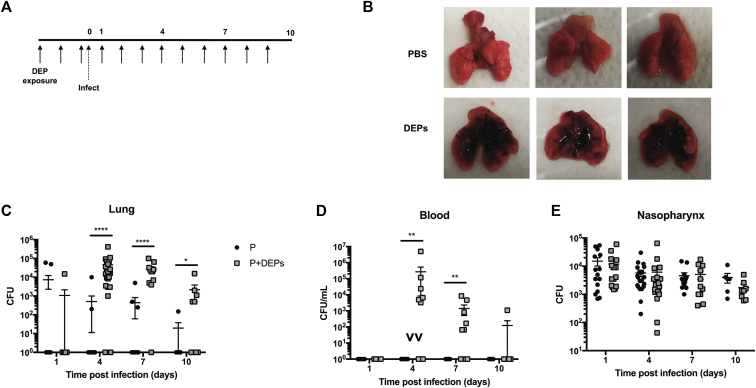
DEP exposure increases susceptibility to invasive pneumococcal disease. **A,** Experimental outline. Female CD1 mice were exposed daily to either PBS or DEPs via the intranasal route. After the third exposure, mice were infected with 10^5^ CFU of *S pneumoniae* to induce stable nasopharyngeal carriage and daily particulate exposure (or PBS control) was continued for the duration of the experiment. **B,** Lungs of PBS-exposed (*top*) and DEP-exposed (*bottom*) mice at day 7 postinfection. Lung **(C)**, blood **(D)**, and nasopharyngeal **(E)** CFU counts at 1, 4, 7, and 10 days postinfection are shown. Black circles indicate mice subjected to pneumococcal infection only (P), gray squares indicate pneumococcus-infected mice subjected to daily DEP exposure (P+DEPs). Error bars indicate the SEM; ****P < .0001; **P < .01; *P < .05; n = 7 to 21 per group, per time point.

**Fig E1:**
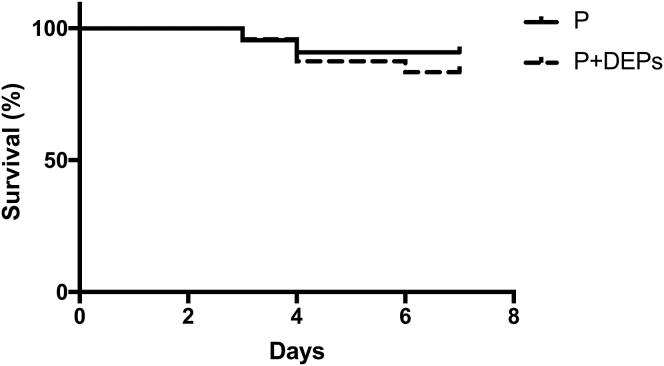
Survival of PBS-exposed (P) and DEP-exposed (P+DEP) mice following pneumococcal carriage infection (n = 24).

In our experiments, CD1 mice were exposed to 80 μg of DEPs in 40 μL of PBS once daily for 3 days before they were infected with *S pneumoniae* (serotype 2, strain D39) and then continuing daily for the duration of the experiment (Fig 1, *A*). Mice were infected with 10^5^ CFU of D39 in 10 μL of PBS to induce asymptomatic nasopharyngeal carriage without dissemination to the lower respiratory tract.^50^^,^^51^ Deposition of particulates in the lungs of DEP-exposed mice was clearly visible throughout infection (Fig 1, *B*). DEP-exposed mice had significantly higher bacterial loads in the lung at day 4 postinfection (*P =* .0001), and this trend was continued at day 7 (*P =* .0001) and day 10 postinfection (*P =* .05, Fig 1, *C*). Lung bacterial loads between 10^3^ and 10^7^ CFU were reported in 90% of DEP-exposed mice (20 of 22) at day 4 postinfection and 90% of mice (10 of 11) at day 7 postinfection. The mean values for these time points were 4.6 × 10^4^ and 2.7 × 10^4^ CFU, respectively. In contrast, only 10% to 20% of sham-treated mice had pneumococci in their lungs at these time points. By day 10 postinfection, only 55% of DEP-exposed mice (5 of 9) had pneumococci in their lungs and the mean bacterial load was 2.2 × 10^3^ CFU, suggesting that DEP-exposed mice retain the capacity to clear infection from their lungs over time. In addition, DEP-exposed mice developed bacteremia by day 4 postinfection, whereas the controls did not (Fig 1, *D*). Despite the association between DEP exposure and high lung and/or blood bacterial loads, there was no significant difference in nasopharyngeal bacterial load compared with that in PBS-treated mice (Fig 1, *E*), nor was there a difference in survival time (*P =* .467, see Fig E1 in this article’s Online Repository at www.jacionline.org).

### DEP exposure is associated with lung inflammation

**Fig 2:**
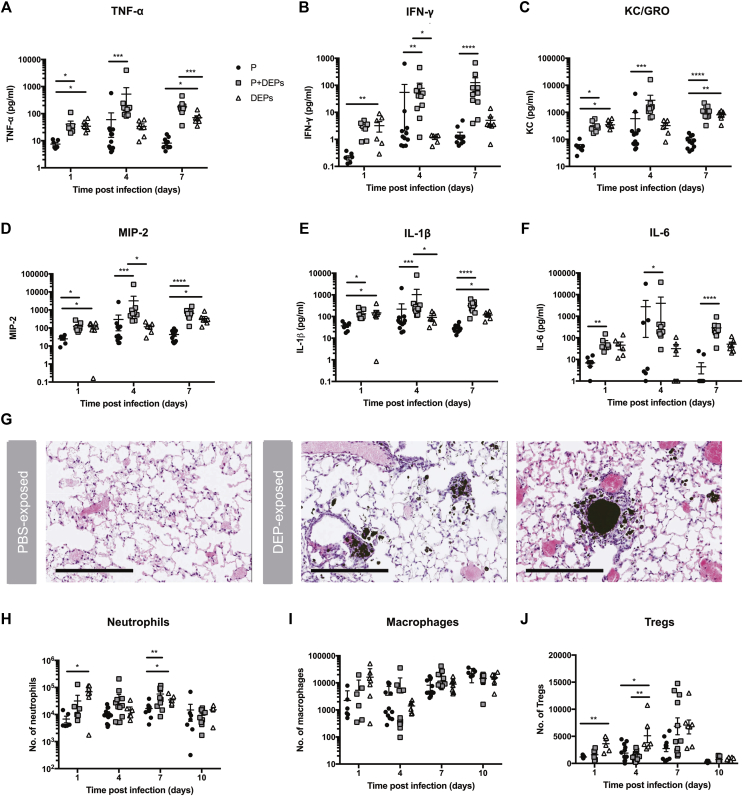
DEP exposure drives early inflammation in the lungs that is exacerbated in the context of infection. **A-F,** TNF-α, IFN-γ, keratinocyte chemoattractant/human growth-regulated oncogene (KC/GRO), macrophage inflammatory protein 2 (MIP-2), IL-1β, and IL-6 levels in lung homogenate were measured for mice subjected to pneumococcal infection only (P, *black circles*), pneumococcus-infected mice subjected to daily DEP exposure (P+DEPs, *gray squares*), and mice subjected to daily DEP exposure in the absence of infection (DEPs, *white triangles*). **G,** Representative images of lungs from sham-exposed (*left*) and DEP-exposed (*right*) mice at day 7 postinfection following hematoxylin and eosin staining. **H-J,** The number of neutrophils (CD45^+^Gr1^+^), macrophages (CD45^+^F4/80^+^CD11b^+^), and regulatory T cells (Tregs) (CD45^+^CD4^+^Foxp3^+^) in lung cell suspension was measured by flow cytometry for mice subjected to pneumococcal infection only (P, *black circles*), pneumococcus-infected mice subjected to daily DEP exposure (P+DEPs, *gray squares*) and mice subjected to daily DEP exposure in the absence of infection (DEPs, *white triangles*). The gating strategy is shown in Fig E2. Error bars indicate the SEM; ****P < .0001; ***P < .001; **P < .01; * *P* < .05. Scale bars represent 200 microns, n = 7 to 10 per group, per time point.

**Fig E2:**
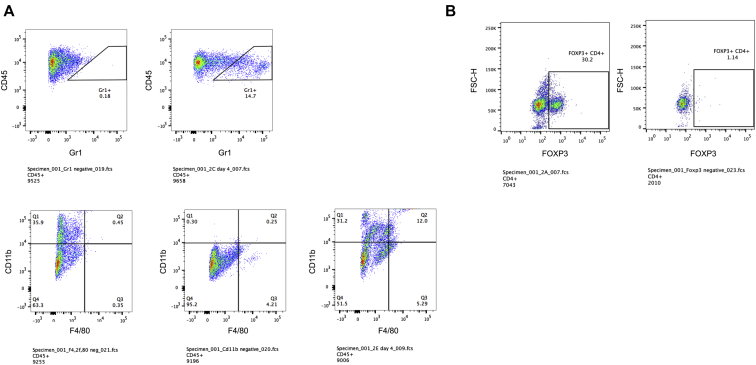
Gating strategy for neutrophils (CD45^+^Gr1^+^), macrophages (CD45^+^F4/80^+^CD11b^+^) **(A)** and Tregs (CD45^+^ CD4^+^ Foxp3^+^) **(B)** in lung cell suspensions.

DEPs were inflammatory in the absence of pneumococcal infection, leading to significantly increased levels of TNF-α, IFN-γ, keratinocyte chemoattractant/human growth-regulated oncogene (KC/GRO), macrophage inflammatory protein 2, and IL-1β compared with the levels in pneumococcus-infected control mice at day 1 postinfection (Fig 2, *A*-*E*). By day 4 and 7 postinfection, however, the DEP-exposed, pneumococcus-infected mice had higher levels of these proinflammatory cytokines than the uninfected mice exposed only to DEP, and both groups had higher levels of these cytokines compared to the sham-treated pneumococcus-infected controls (Fig 2 *A-F*). These data suggest that DEP exposure further exacerbates proinflammatory cytokine production during active lung infection. IL-10, IL-12p70, and IL-17A were also included in the Meso Scale Discovery cytokine array; however, low levels of IL-10 and IL-12 were measured at all time points, with higher titers of IL-17A measured at day 7 only (data not shown). The proinflammatory nature of DEP exposure alone is also apparent from lung histology, with marked influx of leukocytes surrounding DEPs deposited within the lung tissue (Fig 2, *G*). As expected, the majority of DEP deposition was in close proximity to the respiratory airways. DEP exposure was accompanied by neutrophil infiltration into the lungs at day 1 and day 7 postinfection (Fig 2, *H*). There was no significant difference in the number of macrophages at any time point (Fig 2, *I*); however, there was an increase in the number of regulatory T cells in the lungs of DEP-exposed mice compared with the number in the lungs of the sham-treated mice at day 1 and 4 postinfection (Fig 2, *J*), presumably to counteract the proinflammatory response (see Fig E2 in this article's Online Repository at www.jacionline.org for gating strategy).^52^ These proinflammatory responses in the lung are likely to cause significant tissue damage, permitting the bacterium to translocate into the blood stream.^46^^,^^52^^,^^53^

### Alveolar macrophages become congested with DEPs, which reduces their phagocytic function and leads to increased production of proinflammatory cytokines

**Fig 3:**
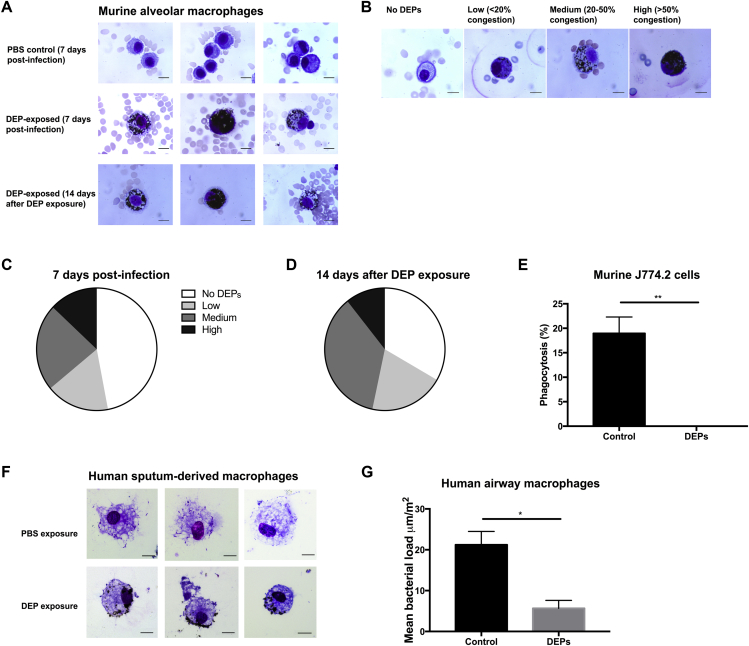
DEP-congested alveolar macrophages have reduced phagocytic ability. **A,** Alveolar macrophages isolated by BAL from PBS- (*top*) and DEP- (*middle*) exposed mice 7 days postinfection. Alveolar macrophages isolated from DEP-exposed mice 14 days after the last DEP exposure (*bottom*), suggesting that clearance of DEPs from macrophages is a slow process. **B,** Scoring guide for alveolar macrophages according to how congested with DEPs they are. No uptake of DEPs, low (<20% of cell cytoplasm is congested with DEPs), medium (20% to 50% congestion), and high (>50% congestion) uptake were scored as shown. **C,** Proportion of alveolar macrophages graded as characterized by low, medium, or high congestion at day 7 postinfection following daily DEP exposure. **D,** Proportion of alveolar macrophages graded as characterized by low, medium, or high congestion 14 days after the last DEP exposure. **E,** Pneumococcal opsonophagocytic ability of mouse J774.2 macrophages treated with either PBS (control) or DEPs. **F,** Uptake of DEPs (*bottom*) versus control (PBS-treated) airway macrophages isolated from sputum of human volunteers. **G,** Phagocytosis of pneumococci by human airway macrophages pretreated with either PBS (control) or DEPs. Error bars indicate the SEM; ***P < .001; **P < .01; *P < .05. For each experimental condition, n = 3 to 5.

**Fig E3:**
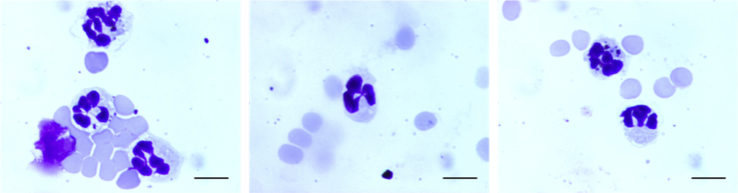
Characterization of neutrophils in BAL fluid of DEP-exposed mice. Cells isolated in BAL fluid were Giemsa-stained, and neutrophils were identified by their lobed nucleus. Scale bars represent 10 microns.

Despite a robust inflammatory response and increased numbers of lung neutrophils (Fig 2), the host is unable to clear *S pneumoniae* from the lungs following DEP exposure. Macrophages and neutrophils are essential for clearance of pathogens and debris in the lungs,^54^^,^^55^ and reduction of alveolar macrophage phagocytic function is known to permit uncontrolled replication of the pneumococcus in the lungs.^56^^,^^57^ We hypothesized that the pulmonary phagocytes become congested with particulate following DEP exposure, which could reduce their phagocytic ability and thus impair bacterial clearance. To test this, airway macrophages and neutrophils were isolated by BAL from mice exposed to DEPs for 7 days (Fig 3, *A*). More than half of the alveolar macrophages (53%) were congested with DEPs; a small proportion of macrophages (13%) were heavily congested with DEPs (as defined in Fig 3, *B*), whereas a further 23% and 17% had medium and low DEP congestion, respectively (Fig 3, *C*). Interestingly, this observation appeared to be unique to airway macrophages, as neutrophils were not congested with DEPs (see Fig E3 in this article’s Online Repository at www.jacionline.org). This may suggest that macrophages are the most important phagocytic cell for DEP clearance, that neutrophils are capable of rapidly clearing ingested particulates, or it may simply reflect the short life span and rapid turnover of neutrophils.^58^ To determine how quickly DEPs are cleared from airway macrophages, mice were exposed to DEPs for a total of 3 consecutive days and BAL fluid was obtained 1, 3, 7, or 14 days after the last DEP exposure. Even after 14 days, the majority of airway macrophages (67%) were still congested with DEPs (Fig 3, *D*), suggesting that DEP exposure may affect macrophage function for prolonged periods, potentially leaving the host susceptible to a variety of respiratory infections.

**Fig E4:**
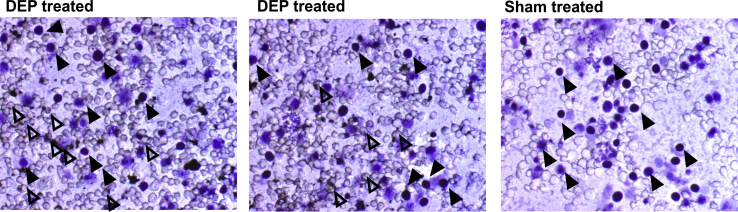
Viability of J774.2 cells is not affected by DEP exposure. J774.2 macrophages were treated for 2 hours with either DEPs (*left, middle*) or sham-treated (*right*) and stained with Trypan blue to confirm that DEP exposure does not affect viability of cells. Dye is taken up by a minority of cells (*closed arrows*), which indicates cell death. DEP uptake by J774.2 macrophages is also visible (*open arrows*). Scale bars represent 10 microns.

To determine whether particulate congestion could impair bacterial phagocytosis by airway macrophages, we performed OPKAs using mouse J774.2 macrophage cells. Cells were pre-exposed to DEPs for 2 hours before addition of opsonized D39. Viable counts were determined following overnight growth on blood agar base plates. Under these conditions, sham exposure resulted in 17% pneumococcal phagocytosis; however, pneumococcal clearance was completely ablated following DEP exposure (Fig 3, *E*). There was no difference in viability of DEP- and sham-exposed cells (see Fig E4 in this article’s Online Repository at www.jacionline.org), suggesting that the altered phagocytic ability was not due to a reduction in cell viability following DEP exposure. We also performed a similar phagocytic killing assay with induced sputum macrophages isolated from healthy human volunteers. Uptake of DEPs by these macrophages was observed (Fig 3, *F*), and pneumococcal phagocytosis was significantly impaired compared with that by PBS-exposed macrophages (Fig 3, *G*). These data suggest that the impaired innate immune mechanisms observed in our mouse model are also at play in humans.

**Fig 4:**
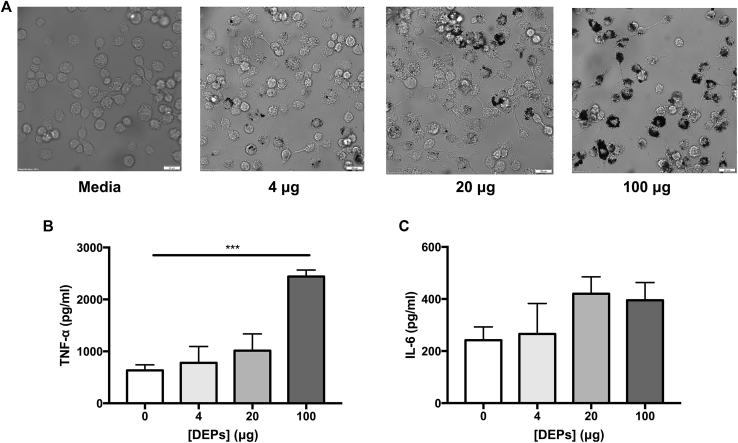
DEP exposure increases proinflammatory cytokine secretion by BMDMs. Murine BMDMs were treated with 4, 20, or 100 μg of DEPs. After 24 hours of exposure, the cells were washed to remove DEPs from the culture. The BMDMs were stimulated with D39 on day 6 following DEP exposure. IL-6 and TNF-α production was measured by ELISA. **A,** Uptake of DEPs (4, 20, or 100 μg) by murine BMDMs following 24-hour treatment. **B-C**, TNF-α and IL-6 in culture supernatant 24 hours after pneumococcal stimulation (normalized against total protein production). Results represent 4 independent experiments. Bars indicate mean cytokine production; error bars represent the SEM; ***P < .001; n = 4 per condition).

We also sought to determine the effect of DEP exposure on proinflammatory cytokine production by macrophages. Murine BMDMs were exposed to DEPs (4, 20, or 100 μg) for 24 hours, after which uptake of DEPs was visible (Fig 4, *A*). Six days later, the BMDMs were stimulated with *S pneumoniae* or medium alone and TNF-α and IL-6 production in culture supernatants was measured by ELISA. Production of TNF-α and IL-6 following stimulation with pneumococci was increased with exposure to higher concentrations of DEPs (Fig 4, *B*-*C*). However, no TNF-α or IL-6 was measured in the culture supernatants of DEP-exposed BMDMs stimulated with medium alone (data not shown). These data suggest that DEP-congested macrophages produce higher levels of inflammatory cytokines following microbial challenge than do unexposed macrophages, which is likely to contribute to the proinflammatory environment of the lung observed in DEP-exposed, pneumococcus-infected mice.

### Pneumococci adhere to DEPs *in vitro*

**Fig 5:**
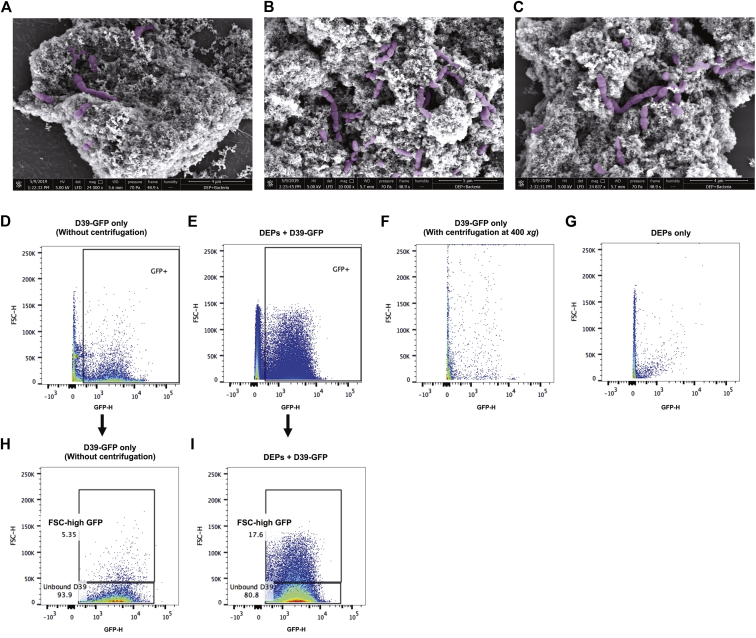
Pneumococci adhere to DEPs *in vitro*. **A-C,** Following coincubation of D39 with DEPs, pneumococci (*highlighted in purple*) can be seen in close proximity to the particulate by scanning electron microscopy. Original images are shown in Fig E6. **D-G,** Flow cytometry plots for D39-GFP without slow-speed centrifugation step **(D)**, coincubated DEPs and D39-GFP after slow-speed centrifugation **(E)**, D39-GFP only after slow-speed centrifugation **(F),** and DEPs alone **(G)** are shown. The percentages of unbound and FSC-high GFP events in **(D)** and **(E)** are displayed in **(H)** and **(I)**, respectively.

**Fig E5:**
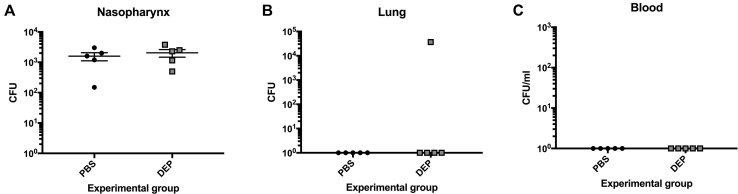
Exposure of mice to DEPs before infection only does not result in bacterial dissemination into the lungs or blood. Mice were exposed to DEPs for 6 days before induction of pneumococcal carriage (no DEP exposure after infection) and culled at day 4 after infection to assess nasopharyngeal **(A)**, lung **(B)**, and blood **(C)** CFUs.

**Fig E6:**
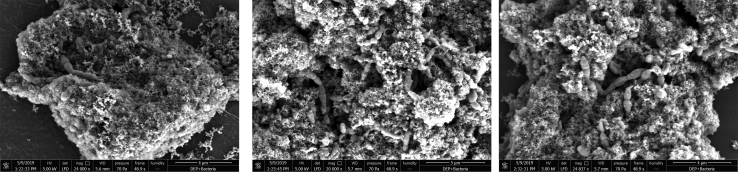
Scanning electron microscopy images showing adherence of pneumococci to DEPs.

The increased lung and blood bacterial loads of DEP-exposed mice described in Fig 1 were apparent when mice were exposed to DEPs on a daily basis both before and after induction of pneumococcal carriage. The same effects were not observed, however, when mice were exposed to DEPs for 6 consecutive days before infection (with no postinfection DEP exposure) (see Fig E5 in this article’s Online Repository at www.jacionline.org), despite being subjected to the same number of DEP exposures (6 in total). We therefore hypothesized that DEPs could be binding to pneumococci as they pass through the nasopharynx, thus dragging bacteria down into the lungs. To determine whether pneumococci could bind DEPs, we incubated D39 or D39-GFP with DEPs on an over-end rotator for 20 minutes. Samples were centrifuged at 400 *g* for 5 minutes to pellet DEPs, and unbound pneumococci were removed with the supernatant. The pellet was washed with PBS and samples were prepared for scanning electron microscopy or flow cytometry analysis. We observed adherence of pneumococci to DEPs by scanning electron microscopy (Fig 5, *A-C*, see Fig E6 in this article's Online Repository at www.jacionline.org for original image) and these observations were confirmed by using flow cytometry (Fig 5, *D-I*). Coincubation of D39-GFP with DEPs led to an increase in the size (FSC) of a proportion of recorded GFP-positive events, suggesting binding of bacteria to the larger DEPs (Fig 5, *D-E*). We also confirmed that centrifugation at 400 *g* does not pellet pneumococci in the absence of DEP adherence (Fig 5, *F*), and we showed that DEPs alone do not fluoresce at this wavelength (Fig 5, *G*). These data suggest that the increase in high-FSC GFP-positive events in the slow-speed centrifugation pellet of coincubated DEPs and D39-GFP (Fig 5, *H-I*) was the result of pneumococci adhering to the particulate. Together, these data suggest that inhaled DEPs can drag pneumococci from the nasopharynx downward into the lower respiratory tract, leading to increased susceptibility to pneumonia and bloodstream infection.

### DEPs sustain survival of pneumococci

**Fig 6:**
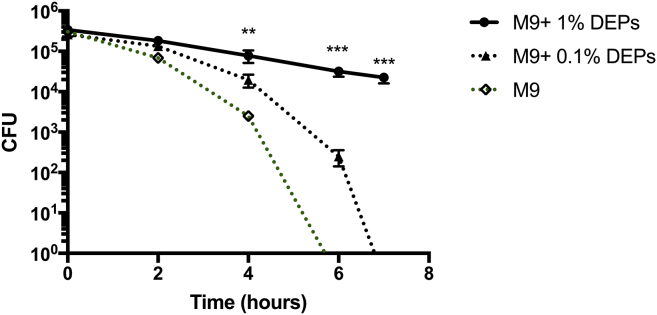
DEPs sustain survival of pneumococci *in vitro*. Viable count for pneumococci in M9 minimal medium supplemented with 1% DEPs (M9 + 1% DEPs, *solid black line*) and 0.1% DEPs (M9 + 0.1% DEPs, *broken black line*) as compared with M9 medium without supplement (M9, *broken green line*) over 7 hours. Graph represents 4 independent experiments.

We also investigated the effect of DEP exposure on pneumococcal survival. Bacteria were incubated in M9 minimal medium supplemented with either 1% or 0.1% DEPs. Viable counts were calculated at 0, 2, 4, 6, and 7 hours after inoculation and compared with the counts with minimal medium without supplement (M9). For the M9 group, the CFU count fell rapidly over time, and by 6 hours no bacteria were detected. In contrast, the CFU count decreased at a substantially slower rate when pneumococci were maintained in minimal medium supplemented with 0.1% or 1% DEPs (M9 + 0.1% or 1% DEPs). The CFU count for the M9 plus 1% DEP group was significantly higher than that of the M9 group at 4, 6, and 7 hours after inoculation (*P* = .0066, .0005, and .0009 respectively, Fig 6). These data suggest that DEPs may provide metabolites for pneumococci, promoting survival within the lung. DEPs contain a mixture of polycyclic aromatic hydrocarbons and nitro-substituted polycyclic aromatic hydrocarbons, among other chemical compounds,^59^ although further analysis is required to identify which components promote survival of the pnemococcus. This is the first report, to our knowledge, demonstrating that pneumococci are able to use environmental pollutants for metabolism.

### Inhaled DEP exposure increases susceptibility to invasive pneumococcal pneumonia in resistant hosts

**Fig 7:**
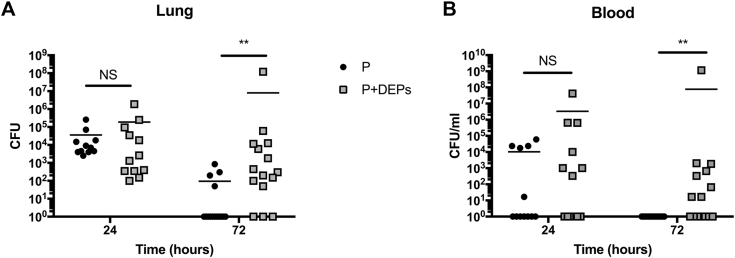
Daily inhalation of DEPs increases lung and blood bacterial loads during pneumococcal pneumonia in resistant mice. BALB/c mice were exposed daily to PBS or DEPs before (and following) infection with pneumococci. Mice were culled at 24 or 72 hours postinfection to assess lung **(A)** and blood **(B)** CFUs.**P < .01; NS = nonsignificant; n = 12 to 15 per group, per time point.

To expand on our previous findings showing that exposure to DEPs increases susceptibility to IPD, we repeated our *in vivo* experiments using a mouse strain normally resistant to pneumococcal pneumonia.^52^^,^^53^ In this model, resistant BALB/c mice were exposed daily to DEPs and lung infection was induced by intranasal infection with 1 × 10^6^ CFU in 50 μL of PBS, meaning that both the DEPs and pneumococci were deposited directly into the lungs. There was no significant difference in lung or blood bacterial loads of DEP-exposed mice compared with those of the sham (PBS)-treated mice at 24 hours postinfection; however, by 72 hours postinfection, the majority of sham-treated mice had cleared the infection, whereas 80% of the DEP-exposed mice (12 of 15) still harbored pneumococci in their lungs (Fig 7, *A*). In addition, 60% of the DEP-exposed mice (9 of 15) had bacteremia at 72 hours postinfection, whereas none of the sham-treated mice had bacteremia at this time point (Fig 7, *B*). This recapitulates our earlier findings, showing that when DEPs and pneumococci are both present in the lungs, bacterial clearance is impaired, even in pneumonia-resistant mouse strains, which normally have robust bacterial clearance mechanisms in place.

## Discussion

The results described here show that inhaled exposure to DEPs alters pneumococcal interactions with its host, promoting transition from asymptomatic nasopharyngeal commensal to potentially life-threatening pathogen. During pneumococcal carriage, the pneumococcus is ordinarily restricted to the nasopharynx and progression to the lungs is rare.^49^ However, DEP exposure significantly increases the bacterial load in the lung, with 90% of DEP-exposed mice displaying high bacterial loads at day 4 and 7 postinfection. This increase in lung CFU burden leads to bacteremia in approximately a third of mice by day 4 and half by day 7 postinfection. Surprisingly, despite bacteremia, there was no significant difference in the survival of DEP-exposed mice compared with that of the control mice. There was also no significant difference in bacterial load within the nasopharynx at any time point, suggesting that the major effect of DEP inhalation is due to events within the lower airways (ie, the creation of a proinflammatory environment within the lungs, combined with the congestion of airway macrophages). In our *in vivo* experiments, we mimicked ambient air levels of fine PM recorded in cities across Western Europe (∼20 μg/m^3^ per day).^60^ The theoretical exposure for people living in these areas is 200 mg per day, assuming a daily intake of 10,000 m^3^ of air for an average adult, which is an approximately equivalent weight/volume ratio for a 30-g mouse compared to a 75-kg human (2.67 μg DEPs per gram body mass). Although only a proportion of PM may reach the lungs because of various clearance mechanisms (eg, mucus and cilia), meaning that the experimental exposure presented in this article may be closer to much higher levels of PM, these findings suggest that under the right circumstances (dependent on age, genetics, and coinfection status), exposure to environmental particulates such as DEPs could significantly alter the outcome of pneumococcal carriage, increasing susceptibility to invasive disease in humans.

Our data suggest that DEP exposure causes an early increase (day 1 postinfection) in lung proinflammatory cytokine production relative to that in PBS-treated mice. The inflammatory environment is exacerbated by active lung infection (of the DEP-exposed, pneumococcus-infected mice) at day 4 and 7 postinfection. This proinflammatory response is likely to cause substantial tissue damage, permitting the bacterium to access the blood stream.^46^^,^^52^^,^^53^ This is corroborated by the lung histology images, which show a substantial influx of leukocytes to areas with high DEP deposition, and by our flow cytometry data, which also show increased neutrophil influx compared with that in sham-treated mice at multiple time points. These observations are supported by previous reports, which found that DEP inhalation causes influx of both neutrophils and macrophages to the lung and increased expression of IL-4, IL-6, IL-13, TNF-α, and NF-κB by lung cells, as well as an upregulation of mucus production and deposition of collagen and elastic fibers in the alveolar septum.^37^^,^^61^ These data suggest that tissue remodeling occurs following DEP-driven inflammation. There is also a strong body of evidence to suggest that DEP inhalation can increase lung inflammation and bronchial hyperresponsiveness caused by house dust mite and soybean allergen.^39^^,^^41^^,^^42^ These data suggest that DEP exposure may exacerbate allergic airway inflammation, generating a mixed T_H_2/T_H_17 response.^39^^,^^41^^,^^42^ Exposure to DEPs also resulted in increased levels of markers for both allergic (eosinophilia and IL-5 production) and nonallergic (macrophage chemoattractant protein-1, IL-8, and neutrophilia) inflammation in atopic human volunteers.^40^ Alarmingly, DEP exposure has also been shown to alter the gene expression profile of human bronchial epithelial cells *in vitro*, with alterations in genes associated with metabolism of lipids and xenobiotics, as well as changes in inflammatory cytokines such as IL-1β,^62^ suggesting that exposure to pollutants could have far-reaching and long-lasting consequences.

We observed that DEPs are adhesive by nature, and we showed that pneumococci bind to DEPs following *in vitro* incubation. It is possible that DEPs adhere to pneumococci as they pass through the nasopharynx, thereby pulling bacteria down into the lung. In support of this, we did not observe pneumococci in the lungs of mice that were exposed to DEPs only before infection. These mice were subjected to the same number of DEP exposures as those in Fig 1 (6 in total); however, exposure was restricted to preinfection. It is reasonable to assume therefore that this phenomenon of bacterial transfer to the lower respiratory tract following adherence to PM may also occur in human carriage, as airborne DEPs mix with moisture in the nasal cavity as we breathe in. This effect does not appear to alter the bacterial load within the nasopharynx, presumably because only small numbers of planktonic pneumococci (those which are not strongly attached to host cells within the nasopharynx) are transferred to the lungs. Upon reaching the lungs, the pneumococci are able to replicate, reaching the higher CFU levels reported here. We also demonstrate that pneumococci can utilize DEPs as a source of metabolites in the absence of other energy sources, so it is possible that the daily administration of DEPs in our *in vivo* model is aiding bacterial survival within the lungs. This is the first report, to our knowledge, demonstrating that pneumococci are able to use environmental pollutants as a source of metabolites.

However, the ability of DEPs to adhere to pneumococci and utilize DEPs as a source of metabolites is unlikely to be the sole reason for progression toward IPD. We show that airway macrophages become congested with DEPs following intranasal exposure, and that this reduces their phagocytic function, which would permit increased replication of the bacterium in the lungs given the key role of alveolar macrophages in host anti-pneumococcal defense.^56^^,^^57^ We also found that DEP exposure reduces the phagocytic ability of human airway macrophages, suggesting that impaired bacterial clearance by airway macrophages following inhaled particulate exposure may also potentially affect pneumococcal infection outcome in humans. Of course, there are differences in phenotype between induced sputum macrophages and alveolar macrophages from the more distal airways^63^; however, both macrophage populations exhibited significant impairment in phagocytosis following exposure to inhaled DEPs which would clearly contribute to susceptibility to pneumococcal disease. Congestion of airway macrophages and reduced phagocytosis following exposure to DEPs has been reported in other infection models, such as *P aeruginosa* and *L monocytogenes*.^43^^,^^44^ Our data build on these observations, showing that exposure to DEPs at levels equivalent to the levels of fine PM measured in cities across Western Europe, can alter pneumococcal infection outcome, promoting a switch from asymptomatic nasopharyngeal carriage to dissemination to the lungs and blood, thus tipping the balance toward invasive disease. Together, these studies suggest that exposure to DEPs can alter host-pathogen responses for a range of respiratory bacteria.

In addition to phagocytosis of bacteria, airway macrophages play a critical role in removing apoptotic and necrotic cells while also controlling neutrophil infiltration and cytokine production in the lungs during pneumococcal pneumonia.^64^ This might explain the increase in proinflammatory cytokines in DEP-exposed, infected mice relative to the level in sham-treated mice, as congested alveolar macrophages are unable to control neutrophil influx and proinflammatory cytokine production. Indeed, our flow cytometry data show that there is an increase in neutrophils both early on (day 1) and later (day 7) during infection in the DEP-exposed mice.

We found that even 2 weeks after the last DEP exposure, airway macrophages remain congested with DEPs, suggesting that DEP clearance by macrophages themselves and/or clearance of congested macrophages by other cells is a slow process. These data suggest that DEP exposure may have a long-term effect on the phagocytic function of airway macrophages, potentially leaving the host susceptible to a variety of respiratory infections. In addition, we show that DEP congestion leads to increased TNF-α and IL-6 secretion by BMDMs in response to pneumococcal challenge. Taken together with the clearance data, this suggests that long-term congestion of airway macrophages could lead to prolonged lung inflammation and elevated risk of pneumococcal disease even weeks or months following DEP exposure. In the context of pneumococcal pneumonia, uncontrolled lung inflammation can grant the pneumococcus access to the blood stream, which can lead to bacteremia and sepsis.^52^ It is likely that the combination of reduced airway macrophage phagocytic function and increased inflammation observed in our model allows the bacterium to replicate to high numbers in the lung and permits the pneumococcus to access the blood stream. Our experiments using a pneumococcal pneumonia model in resistant (BALB/c) hosts recapitulate these findings, showing that when DEPs and pneumococci are both present in the lungs, bacterial clearance is impaired, probably mainly due to reduced function of alveolar macrophages, which are key in controlling pneumococcal infection,^57^^,^^64^^,^^65^ and that DEPs are able to sustain bacterial survival and proliferation.

In summary, we show that daily inhaled exposure to DEPs disrupts asymptomatic nasopharyngeal carriage in mice and promotes pneumococcal dissemination to the lungs and blood. Our results show that pneumococci are transported from the nasopharynx to the lungs following adherence to DEPs and are able to utilize DEPs as a source of metabolites, promoting survival within the lungs. We demonstrate that inhaled DEPs lead to influx of neutrophils in the lungs, even in the absence of infection. However, airway macrophages (isolated from both mouse and human), which are key for control of pneumococcal infection, become congested with DEPs, which significantly reduces their phagocytic function while promoting a proinflammatory environment within the lungs. This results in increased pneumococcal loads in the lungs and associated lung tissue inflammation leading to bacterial entry into the bloodstream. These data provide compelling evidence that exposure to particulate pollutants such as DEPs can alter the relationship between the host and pneumococcus, tipping the balance toward invasive pathogenicity. Given that 91% of the world’s population inhabit areas where the air pollution exceeds World Health Organization guideline limits,^19^ reducing the amount of airborne pollution has the potential to reduce the global burden of pneumococcal disease significantly. A recent global assessment of the effect of environmental pollution on health commissioned by the World Health Organization found that 35% of lower respiratory tract infections are due to modifiable environmental factors.^66^ Such modifications would include reducing airborne pollution.^66^ Therefore, our data add impetus to coordinate global efforts to reduce airborne pollution and transition toward more sustainable energy sources that produce cleaner air.

**Key messages**

• Daily inhaled exposure to DEPs increases susceptibility to pneumococcal disease by disrupting the delicate balance that exists in the nasopharynx during asymptomatic carriage.
• DEPs cause lung inflammation and is readily ingested by airway macrophages, which significantly reduces their phagocytic function, leading to increased pneumococcal loads within the lungs and translocation into blood.
